# Efficacy and safety of modified *Xiaoyao-san* in the treatment of postoperative depression of breast cancer: a systematic review and meta-analysis

**DOI:** 10.3389/fphar.2026.1734584

**Published:** 2026-08-28

**Authors:** Bozhen Huang, Hongyi Lan, Meijiao Zhou, Shanshan Song, Manman Zhang, Luyao Wang, Min Jiang

**Affiliations:** 1 Department of Traditional Chinese Medicine, Beijing Shijitan Hospital, Capital Medical University, Beijing, China; 2 Department of Cardiology, Wuxi Hospital of Traditional Chinese Medicine, Wuxi, China; 3 Department of Rheumatology, First Teaching Hospital of Tianjin University of Traditional Chinese Medicine, Tianjin, China; 4 Institute of Basic Theory for Chinese Medicine, China Academy of Chinese Medical Sciences, Beijing, China

**Keywords:** breast cancer, depression, meta-analysis, randomized controlled trial, systematic review, *Xiaoyao-san*

## Abstract

**Objective:**

To comprehensively evaluate the efficacy and safety of modified *Xiaoyao-san* (XYS) in the treatment of postoperative depression of breast cancer patients through systematic review and meta-analysis.

**Methods:**

Randomized controlled trials (RCTs) on the treatment of postoperative depression of breast cancer patients with modified XYS were searched from eight databases. All relevant literature from the inception of each database up to 12 July 2026 were retrieved. The Cochrane risk of bias assessment tool was used to evaluate the risk of bias in all included studies. After screening and data extraction, RevMan 5.2 and Stata 12.0 were used for meta-analysis.

**Results:**

11 RCTs were included, involving a total of 748 patients, with 374 in the treatment group and 374 in the control group. Subgroup analyses showed that modified XYS plus antidepressants (AD) was associated with a significantly greater improvement in depressive symptoms than AD alone [SMD = −0.99, 95% CI (−1.57, −0.42), *p* = 0.001], whereas modified XYS alone did not significantly improve depressive symptoms compared with the control group [SMD = 0.07, 95% CI (−0.08, 0.23), *p* = 0.358]. In addition, both modified XYS plus AD and modified XYS alone significantly increased 5-HT, DA, and NE levels (*p* < 0.05).

**Conclusion:**

This study indicates that modified XYS has a positive effect on the improvement of depression scale scores, neurotransmitters in patients with postoperative depression of breast cancer. However, further verification is needed through more high-quality RCTs.

**Systematic Review Registration:**

https://www.crd.york.ac.uk/prospero, identifier CRD420251035045.

## 1 Introduction

According to the latest global cancer statistics, breast cancer is the cancer with the highest incidence rate among women worldwide (Bray et al., 2024). Currently, surgery is the core treatment method in breast cancer treatment. For most types of breast cancer, surgery is a crucial step to achieve radical cure or effective local disease control (Czajka and Pfeifer, 2025). While surgery brings hope for survival to breast cancer patients, postoperative changes in physical features, hormonal fluctuations, and fear of death can lead to immense psychological stress for these patients, making them prone to developing depression (Ahn and Suh, 2023; Javan Biparva et al., 2023; Phoosuwan and Lundberg, 2023). The incidence of depression among breast cancer patients worldwide is 32.2% (Pilevarzadeh et al., 2019), and the incidence of breast cancer combined with depression ranks third among all cancers (Golden-Kreutz and Andersen, 2004). Research has found that breast cancer mastectomy is significantly positively correlated with the onset of depression (Zhang Q. et al., 2024), and clinical verification has shown that treating postoperative depression of breast cancer significantly helps improve patients’ overall quality of life and increase long-term survival rates (Setyowibowo et al., 2022). Therefore, actively and effectively treating postoperative depression of breast cancer is an important issue that urgently needs to be addressed.

Among patients with postoperative depression after breast cancer surgery, higher preoperative tumor stage corresponds to more severe postoperative depressive symptoms. (Navari et al., 2008). Currently, commonly used antidepressants in clinical practice can alleviate depressive symptoms, but they may also cause new adverse reactions that affect patients’ quality of life, such as nausea, tachycardia, and constipation (Aapro and Cull, 1999). Research indicates that traditional Chinese medicine can alleviate depressive symptoms in patients with postoperative depression of breast cancer while causing fewer adverse reactions; however, no specific formula recommendations have been proposed (Wang et al., 2023).

Existing meta-analysis studies have found that the traditional Chinese medicine formula *Xiaoyao-san* (XYS) can improve depression, including depression caused by pregnancy, stroke, and other conditions (Wang D. et al., 2022; Liu et al., 2025). The traditional Chinese medicine formula XYS is a classic prescription with liver-soothing and spleen-strengthening effects, documented in the Song Dynasty’s *Tai Ping Hui Min He Ji Ju Fang*. The core composition of XYS as Bupleurum chinense DC. [Apiaceae; Bupleuri Radix et Rhizoma], Paeonia lactiflora Pall. [Paeoniaceae; Paeoniae Radix Alba], Angelica sinensis (Oliv.) Diels [Apiaceae; Angelicae Sinensis Radix], Poria cocos (Schw.) Wolf [Polyporaceae; Poria], Atractylodes macrocephala Koidz. [Asteraceae; Atractylodis Macrocephalae Rhizoma] and Glycyrrhiza uralensis Fisch. [Fabaceae; Glycyrrhizae Radix et Rhizoma]. Modern practitioners often use it as a foundation, modifying it appropriately to create modified XYS for clinical application (Pan et al., 2023). All modified XYS prescriptions extracted from included studies contain these six core botanical drugs. Despite its long history of clinical use, the efficacy and safety of modified XYS in treating postoperative depression of breast cancer remain unclear. This study systematically evaluates the efficacy and safety of modified XYS to provide evidence-based support for its use in this therapeutic context.

## 2 Methods

This systematic review and meta-analysis followed the Preferred Reporting Items for Systematic Reviews and Meta-Analysis (PRISMA) guidelines (Page et al., 2021). The protocol is registered with PROSPERO (Identifier: CRD420251035045).

### 2.1 Inclusion criteria

#### 2.1.1 Types of patients

Patients diagnosed with breast cancer through pathological examination, who underwent surgical treatment for breast cancer, and subsequently developed depression. In this study, postoperative depression was defined as depressive symptoms or depressive disorders occurring after breast cancer surgery and assessed using validated depression assessment instruments, including the Hamilton Depression Rating Scale (HAMD), the Self-Rating Depression Scale (SDS), or other standardized depression-related scales. The postoperative period included both the recovery phase after surgery and subsequent postoperative treatment periods.

#### 2.1.2 Types of interventions

For the treatment of breast cancer, conventional antitumor therapies are administered. For the management of depression, modified XYS has been prescribed. Core botanical information and raw material processing procedures of modified XYS are presented in Table 1. This study complies with all relevant provisions of the Pharmacopoeia of the People’s Republic of China.

**TABLE 1:** Core botanical information and raw material processing of modified XYS.

Species name	Family	Medicinal part	Processing method
Bupleurum chinense DC. [apiaceae; bupleuri radix et rhizoma]	Apiaceae	Dried root	Raw
Angelica sinensis (oliv.) diels [apiaceae; angelicae sinensis radix]	Apiaceae	Dried root	Raw
Paeonia lactiflora pall. [Paeoniaceae; paeoniae radix alba]	Ranunculaceae	Dried root	Raw
Atractylodes macrocephala koidz. [Asteraceae; atractylodis macrocephalae rhizoma]	Asteraceae	Dried rhizome	Raw
Poria cocos (schw.) wolf [polyporaceae; poria]	Polyporaceae	Dried sclerotium	Raw
Glycyrrhiza uralensis fisch. [Fabaceae; glycyrrhizae radix et rhizoma]	Fabaceae	Dried root and rhizome	Honey-fried

#### 2.1.3 Types of comparators

For breast cancer treatment, conventional antitumor therapies are employed. For depression management, conventional antidepressants are prescribed, or non-traditional Chinese medicine therapies such as psychological counseling are administered.

#### 2.1.4 Types of outcomes

Primary outcome measure: depression scale scores. Secondary outcome measures: 5-hydroxytryptamine (5-HT), dopamine (DA), norepinephrine (NE).

#### 2.1.5 Type of studies

Randomized controlled trials (RCTs).

### 2.2 Exclusion criteria

1) Duplicate publication; 2) Significant quality issues, including inappropriate choice of statistical tests, inconsistent reporting of effect sizes, and obvious data errors; 3) Incomplete description of outcome data, particularly when authors fail to respond to requests for clarification; 4) Systematic reviews, meta-analyses, review articles, and animal studies; 5) Non-peer-reviewed papers, such as conference proceedings and newspaper articles.

### 2.3 Search strategy

The databases included PubMed, Embase, Web of Science, Cochrane Library, China National Knowledge Infrastructure (CNKI), Wanfang (WF) Database, VIP Database (VIP), and SinoMed (CBM). All relevant literature from the inception of each database up to 12 July 2026 were retrieved. We combined keywords related to breast cancer (e.g., “Breast Neoplasm,” “Breast Tumor,” and “Breast Cancer”) and depression (e.g., “Depression,” “Depressive Symptom,” and “Depressive Disorder”) with keywords related to modified XYS (e.g., “xiaoyao san,” “kami-soyo-san,” and “TJ-24”). The detailed search strategy for all databases is listed in Supplementary Table S1.

### 2.4 Study selection and data extraction

A systematic screening of the literature was performed by two independent reviewers using EndNote 21. The selection process included reviewing titles, abstracts, and full-text articles according to the inclusion and exclusion criteria. If the two independent reviewers disagreed on eligibility, they jointly reviewed the article to reach consensus. If consensus could not be reached, a third reviewer was consulted for arbitration. After confirming the final selected studies, the following information was extracted: 1) Basic information: first author, publication year, country, sample size, age, study duration, disease stage; 2) Treatment group: intervention measures, study outcomes; 3) Control group: control measures, study outcomes; 4) Information related to literature quality assessment.

### 2.5 Risk of bias assessment

Two reviewers independently assessed the quality of the included literature using the tools recommended in the Cochrane version 5.1 manual (Sterne et al., 2019). The assessment consisted of seven levels: 1) Random sequence generation; 2) Allocation concealment; 3) Blinding of participants and personnel; 4) Blinding of outcome assessment; 5) Incomplete outcome data; 6) Selective reporting; 7) Other bias. It assessed each study in terms of three dimensions: high risk of bias, unknown risk of bias, and low risk of bias. Any inconsistencies in the assessments were resolved through discussions with a third reviewer.

### 2.6 Statistical analysis and publication bias

We used RevMan 5.2 and Stata 12.0 for meta-analysis. For binary variables, we used the risk difference (RD) and 95% confidence interval (CI). For continuous outcomes, we used the standardized mean difference (SMD) and 95% CI. We used the *I* ^2^ test to assess heterogeneity. If the heterogeneity test result was *I* ^2^ < 50% and *p* > 0.1, we used a fixed-effects model; otherwise, we used a random-effects model. To ensure the robustness of the results, we performed sensitivity analysis. Publication bias was assessed through funnel plot symmetry, Egger’s test (Egger et al., 1997), and Begg’s test (Begg and Mazumdar, 1994). Observed asymmetry prompted trim-and-fill adjustment for potential missing studies (Duval and Tweedie, 2000).

## 3 Results

### 3.1 Study selection

**FIGURE 1:**
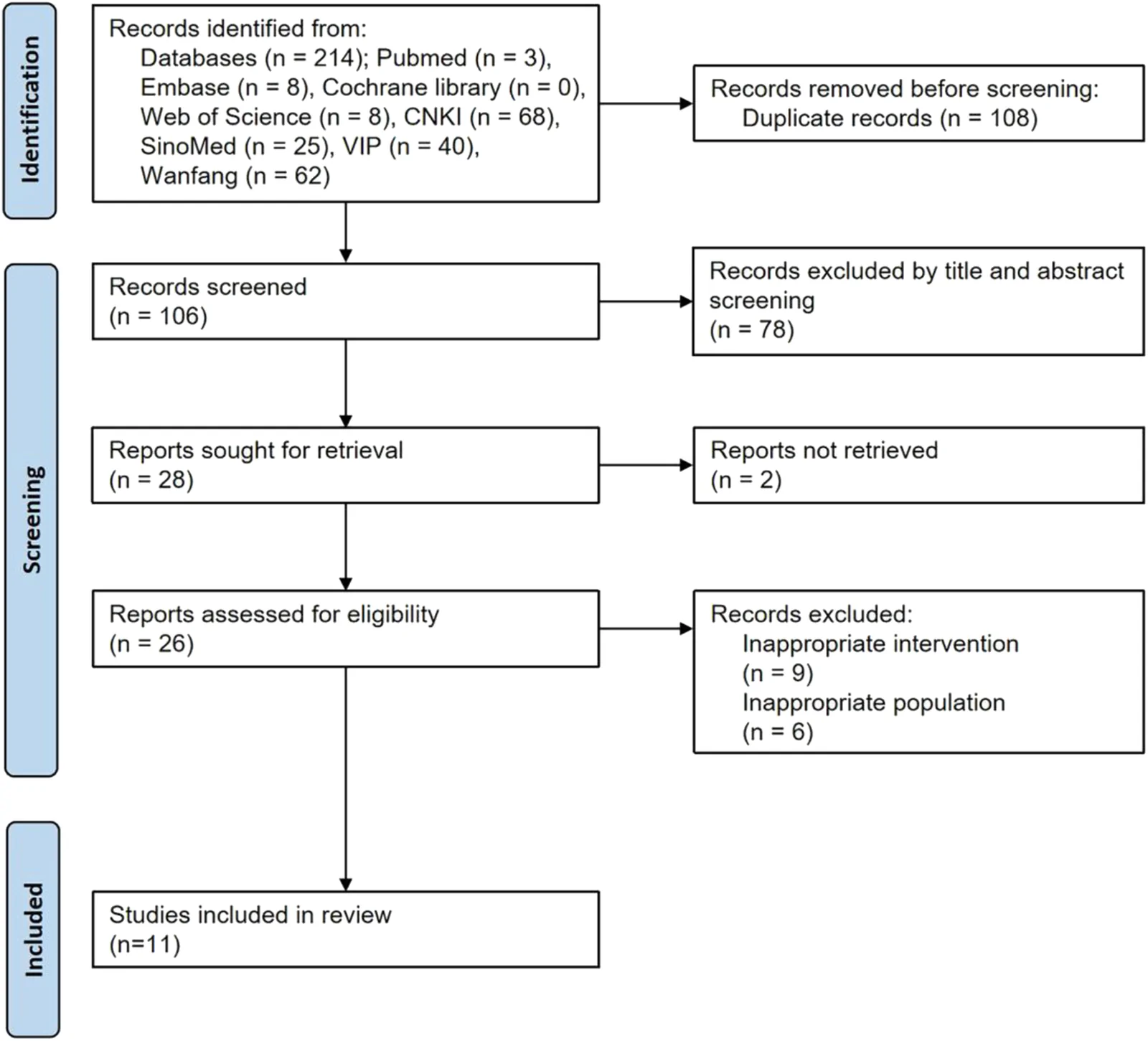
Flow diagram of the selection process of literature.

The initial systematic search across the selected databases yielded 214 records. After the removal of 108 duplicates, 106 records were retained for title and abstract screening. Of these, 78 records were excluded as they did not meet the preliminary inclusion criteria. Full-text articles were assessed for 28 potentially eligible studies, two of which could not be retrieved. A detailed evaluation of the remaining 26 full-text articles led to the exclusion of 15 studies for the following reasons: inappropriate population (*n* = 6) and inappropriate intervention (*n* = 9). Ultimately, 11 studies were included in the meta-analysis. The study selection process is summarized in Figure 1.

### 3.2 Characteristics of included studies

The publication time of the included studies were from 2011 to 2023, with all researches conducted in China. Each study had a sample size ranging from 48 to 100 cases, with a duration of 21–84 days. Breast cancer stages ranged from I to IV. The treatment group included modified XYS, while the control group received conventional antidepressants or usual care, as shown in Table 2.

**TABLE 2:** Basic characteristics of the literature included.

Study ID	Sample size (T/C)	Age	Duration (days)	Diseases stage	Treatment group	Modified XYS	Control group	Outcomes
Gou (2023)	35/35	T: 51.14 ± 5.36 C: 51.28 ± 5.42	56	NR	Modified XYS	Bupleurum chinense DC. [apiaceae; bupleuri radix], 10 g; paeonia lactiflora pall. [Paeoniaceae; paeoniae radix alba], 15 g; angelica sinensis (oliv.) diels [apiaceae; angelicae sinensis radix], 10 g; wolfiporia extensa (peck) ginns [polyporaceae; poria], 10 g; atractylodes macrocephala koidz. [Asteraceae; atractylodis macrocephalae rhizoma], 10 g; glycyrrhiza uralensis fisch. Ex DC. [fabaceae; glycyrrhizae radix et rhizoma], 5 g; astragalus mongholicus bunge [fabaceae; astragali radix], 20 g; codonopsis pilosula (franch.) nannf. [Campanulaceae; codonopsis radix], 20 g; curcuma aromatica salisb. [Zingiberaceae; curcumae radix], 10 g; cyperus rotundus L. [cyperaceae; cyperi rhizoma], 10 g	Alprazolam tablets (0.4 mg, bid)	①②④
Hu et al. (2018)	40/40	T: 47.37 ± 8.60 C: 48.23 ± 8.10	30	NR	Modified XYS + venlafaxine hydrochloride sustained-release tablets (37.5–220 mg/d)	Bupleurum chinense DC. [apiaceae; bupleuri radix], 15 g; paeonia lactiflora pall. [Paeoniaceae; paeoniae radix alba], 15 g; angelica sinensis (oliv.) diels [apiaceae; angelicae sinensis radix], 10 g; wolfiporia extensa (peck) ginns [polyporaceae; poria], 15 g; atractylodes macrocephala koidz. [Asteraceae; atractylodis macrocephalae rhizoma], 15 g; glycyrrhiza uralensis fisch. Ex DC. [fabaceae; glycyrrhizae radix et rhizoma], 10 g; gardenia jasminoides J.Ellis [rubiaceae; gardeniae fructus], 15 g; paeonia suffruticosa andrews [paeoniaceae; moutan cortex], 15 g	Venlafaxine hydrochloride sustained-release tablets (37.5–220 mg/d)	①②③④
Jiao et al. (2019)	24/24	T: 50.50 ± 7.16 C: 47.88 ± 8.44	56	NR	Modified XYS	Bupleurum chinense DC. [apiaceae; bupleuri radix], 10 g; paeonia lactiflora pall. [Paeoniaceae; paeoniae radix alba], 15 g; angelica sinensis (oliv.) diels [apiaceae; angelicae sinensis radix], 10 g; wolfiporia extensa (peck) ginns [polyporaceae; poria], 10 g; atractylodes macrocephala koidz. [Asteraceae; atractylodis macrocephalae rhizoma], 10 g; glycyrrhiza uralensis fisch. Ex DC. [fabaceae; glycyrrhizae radix et rhizoma], 5 g; astragalus mongholicus bunge [fabaceae; astragali radix], 20 g; codonopsis pilosula (franch.) nannf. [Campanulaceae; codonopsis radix], 20 g; curcuma aromatica salisb. [Zingiberaceae; curcumae radix], 10 g; cyperus rotundus L. [cyperaceae; cyperi rhizoma], 10 g	Remifemin tablets (0.28 g, bid)	①
Jin et al. (2016)	41/41	T: 23.8 ± 7.7 C: 22.9 ± 6.9	70	I: 31 II: 39 III: 12	Modified XYS	Bupleurum chinense DC. [apiaceae; bupleuri radix], 9 g; paeonia lactiflora pall. [Paeoniaceae; paeoniae radix alba], 9 g; angelica sinensis (oliv.) diels [apiaceae; angelicae sinensis radix], 6 g; wolfiporia extensa (peck) ginns [polyporaceae; poria], 15 g; atractylodes macrocephala koidz. [Asteraceae; atractylodis macrocephalae rhizoma], 15 g; glycyrrhiza uralensis fisch. Ex DC. [fabaceae; glycyrrhizae radix et rhizoma], 4 g; citrus aurantium L. [rutaceae; aurantii fructus], 15 g; ligusticum chuanxiong hort. [Apiaceae; chuanxiong rhizoma], 9 g; codonopsis pilosula (franch.) nannf. [Campanulaceae; codonopsis radix], 15 g; epimedium brevicornu maxim. [Berberidaceae; epimedii folium], 15 g; curcuma phaeocaulis valeton [zingiberaceae; curcumae rhizoma], 6 g; fritillaria thunbergii miq. [Liliaceae; fritillariae thunbergii bulbus], 6 g; marsdenia tenacissima (roxb.) moon [apocynaceae; marsdeniae tenacissimae caulis], 15 g	Alprazolam tablets (0.4 mg, bid)	①
Shi (2021)	29/29	T: 50.90 ± 8.97 C: 50.79 ± 9.47	42	I: 1 II: 44 III: 11 IV: 2	Modified XYS	Bupleurum chinense DC. [apiaceae; bupleuri radix], 15 g; paeonia lactiflora pall. [Paeoniaceae; paeoniae radix alba], 9 g; angelica sinensis (oliv.) diels [apiaceae; angelicae sinensis radix], 9 g; wolfiporia extensa (peck) ginns [polyporaceae; poria], 9 g; atractylodes macrocephala koidz. [Asteraceae; atractylodis macrocephalae rhizoma], 6 g; glycyrrhiza uralensis fisch. Ex DC. [fabaceae; glycyrrhizae radix et rhizoma], 15 g; mentha canadensis L. [lamiaceae; menthae haplocalycis herba], 6 g; zingiber officinale roscoe [zingiberaceae; zingiberis rhizoma recens], 6 g; acorus tatarinowii schott [acoraceae; acori tatarinowii rhizoma], 6 g; polygala tenuifolia willd. [Polygalaceae; polygalae radix], 9 g; panax ginseng C.A.Mey. [araliaceae; ginseng radix et rhizoma], 6 g	Usual care	①
Sun et al. (2014)	29/29	T: 45.9 ± 11.7 C: 46.4 ± 12.3	42	NR	Modified XYS	Bupleurum chinense DC. [apiaceae; bupleuri radix], 6 g; paeonia lactiflora pall. [Paeoniaceae; paeoniae radix alba], 12 g; angelica sinensis (oliv.) diels [apiaceae; angelicae sinensis radix], 10 g; wolfiporia extensa (peck) ginns [polyporaceae; poria], 15 g; atractylodes macrocephala koidz. [Asteraceae; atractylodis macrocephalae rhizoma], 15 g; glycyrrhiza uralensis fisch. Ex DC. [fabaceae; glycyrrhizae radix et rhizoma], 6 g; citrus medica L. var. Sarcodactylis swingle [rutaceae; citri sarcodactylis fructus], 9 g; codonopsis pilosula (franch.) nannf. [Campanulaceae; codonopsis radix], 15 g; prunella vulgaris L. [lamiaceae; prunellae spica], 20 g; Paris polyphylla sm. [melanthiaceae; paridis rhizoma], 15 g; cremastra appendiculata (D.Don) makino [orchidaceae; cremastrae pseudobulbus], 9 g	Flupentixol and melitracen tablets (1 pill, bid)	①
Sun et al. (2016)	32/32	T: 45.9 ± 11.7 C: 46.4 ± 12.3	42	I ∼ III: 58 IV: 6	Modified XYS	Bupleurum chinense DC. [apiaceae; bupleuri radix], 9 g; paeonia lactiflora pall. [Paeoniaceae; paeoniae radix alba], 12 g; angelica sinensis (oliv.) diels [apiaceae; angelicae sinensis radix], 6 g; wolfiporia extensa (peck) ginns [polyporaceae; poria], 15 g; atractylodes macrocephala koidz. [Asteraceae; atractylodis macrocephalae rhizoma], 15 g; glycyrrhiza uralensis fisch. Ex DC. [fabaceae; glycyrrhizae radix et rhizoma], 9 g; zingiber officinale roscoe [zingiberaceae; zingiberis rhizoma recens], 3 slices; ziziphus jujuba mill. [Rhamnaceae; jujubae fructus], 3 fruits; mentha canadensis L. [lamiaceae; menthae haplocalycis herba], 6 g; rehmannia glutinosa (gaertn.) DC. [orobanchaceae; rehmanniae radix praeparata], 15 g; ligustrum lucidum W.T.Aiton [oleaceae; ligustri lucidi fructus], 15 g; eclipta prostrata (L.) L. [asteraceae; ecliptae herba], 15 g; alisma plantago-aquatica subsp. Orientale (sam.) sam. [alismataceae; alismatis rhizoma], 9 g	Usual care	①②③④
Xiao (2011)	30/30	T: 51.4 ± 8.7 C: 51.3 ± 6.6	42	I ∼ II: 36 III: 24	Modified XYS	Bupleurum chinense DC. [apiaceae; bupleuri radix], 10 g; paeonia lactiflora pall. [Paeoniaceae; paeoniae radix alba], 15 g; angelica sinensis (oliv.) diels [apiaceae; angelicae sinensis radix], 10 g; wolfiporia extensa (peck) ginns [polyporaceae; poria], 20 g; atractylodes macrocephala koidz. [Asteraceae; atractylodis macrocephalae rhizoma], 10 g; glycyrrhiza uralensis fisch. Ex DC. [fabaceae; glycyrrhizae radix et rhizoma], 6 g; prunella vulgaris L. [lamiaceae; prunellae spica], 20 g; Paris polyphylla sm. [melanthiaceae; paridis rhizoma], 15 g; cremastra appendiculata (D.Don) makino [orchidaceae; cremastrae pseudobulbus], 10 g	Flupentixol and melitracen tablets (1 pill, bid)	①
Xiao (2021)	50/50	T: 45.81 ± 11.56 C: 46.37 ± 12.21	84	I: 23 II: 49 III: 21 IV: 7	Modified XYS + paroxetine hydrochloride tablets (20 mg, qd)	Bupleurum chinense DC. [apiaceae; bupleuri radix], 6 g; paeonia lactiflora pall. [Paeoniaceae; paeoniae radix alba], 10 g; angelica sinensis (oliv.) diels [apiaceae; angelicae sinensis radix], 10 g; wolfiporia extensa (peck) ginns [polyporaceae; poria], 15 g; atractylodes macrocephala koidz. [Asteraceae; atractylodis macrocephalae rhizoma], 15 g; glycyrrhiza uralensis fisch. Ex DC. [fabaceae; glycyrrhizae radix et rhizoma], 6 g; mentha canadensis L. [lamiaceae; menthae haplocalycis herba], 6 g; codonopsis pilosula (franch.) nannf. [Campanulaceae; codonopsis radix], 15 g; Paris polyphylla sm. [melanthiaceae; paridis rhizoma], 15 g; rehmannia glutinosa (gaertn.) DC. [orobanchaceae; rehmanniae radix praeparata], 10 g; citrus medica L. var. Sarcodactylis swingle [rutaceae; citri sarcodactylis fructus], 10 g; cremastra appendiculata (D.Don) makino [orchidaceae; cremastrae pseudobulbus], 10 g; prunella vulgaris L. [lamiaceae; prunellae spica], 20 g	Paroxetine hydrochloride tablets (20 mg, qd)	①②③④
Yan et al. (2015)	34/34	51.3 ± 4.8	28	I: 16 II: 33 III: 19	Modified XYS	Bupleurum chinense DC. [apiaceae; bupleuri radix], 10 g; paeonia lactiflora pall. [Paeoniaceae; paeoniae radix alba], 15 g; angelica sinensis (oliv.) diels [apiaceae; angelicae sinensis radix], 15 g; wolfiporia extensa (peck) ginns [polyporaceae; poria], 12 g; atractylodes macrocephala koidz. [Asteraceae; atractylodis macrocephalae rhizoma], 12 g; glycyrrhiza uralensis fisch. Ex DC. [fabaceae; glycyrrhizae radix et rhizoma], 6 g; astragalus mongholicus bunge [fabaceae; astragali radix], 15 g; codonopsis pilosula (franch.) nannf. [Campanulaceae; codonopsis radix], 12 g; albizia julibrissin durazz. [Fabaceae; albiziae flos], 10 g; curcuma aromatica salisb. [Zingiberaceae; curcumae radix], 12 g; mentha canadensis L. [lamiaceae; menthae haplocalycis herba], 8 g; zingiber officinale roscoe [zingiberaceae; zingiberis rhizoma recens], 6 g	Paroxetine hydrochloride tablets (20 mg, qd)	①
Zhang et al. (2015)	30/30	T: 49.2 C: 46.8	21	I ∼ III: 41 IV: 19	Modified XYS + fluoxetine hydrochloride capsules (20 mg, qd)	Bupleurum chinense DC. [apiaceae; bupleuri radix], 15 g; paeonia lactiflora pall. [Paeoniaceae; paeoniae radix alba], 12 g; angelica sinensis (oliv.) diels [apiaceae; angelicae sinensis radix], 15 g; wolfiporia extensa (peck) ginns [polyporaceae; poria], 15 g; atractylodes macrocephala koidz. [Asteraceae; atractylodis macrocephalae rhizoma], 12 g; glycyrrhiza uralensis fisch. Ex DC. [fabaceae; glycyrrhizae radix et rhizoma], 6 g; ziziphus jujuba var. Spinosa (bunge) hu ex H.F.Chow [rhamnaceae; ziziphi spinosae semen], 30 g; gardenia jasminoides J.Ellis [rubiaceae; gardeniae fructus], 9 g; salvia miltiorrhiza bunge [lamiaceae; salviae miltiorrhizae radix et rhizoma], 20 g; citrus reticulata blanco [rutaceae; citri reticulatae viride pericarpium], 12 g	Fluoxetine hydrochloride capsules (20 mg, qd)	①

T, treatment group; C, control group; NR, not reported; ① represents the depression scale scores; ② represents the 5-hydroxytryptamine; ③ represents the dopamine; ④ represents the norepinephrine.

### 3.3 Quality assessment of included studies

**FIGURE 2:**
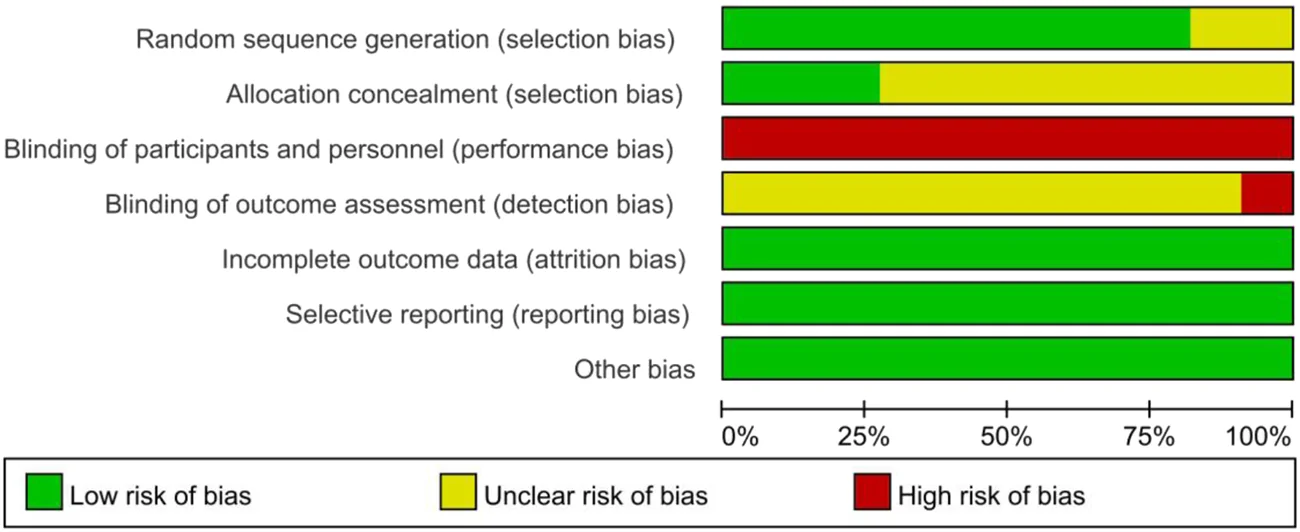
Risk of bias graph for all included studies.

**FIGURE 3:**
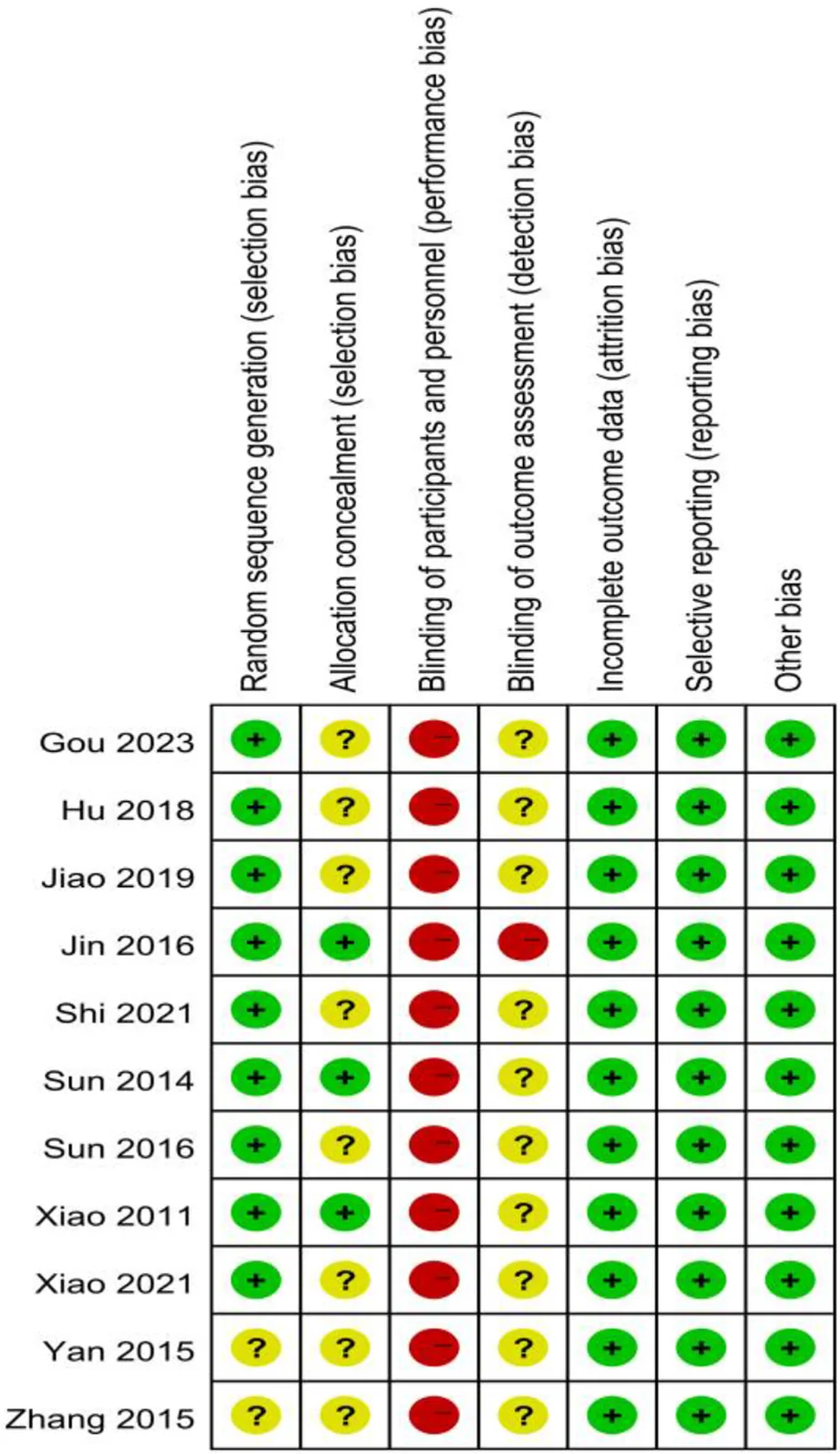
Risk of bias summary for all included studies.

Nine studies detailed methods for generating random sequences (Xiao, 2011; Sun et al., 2014; Sun et al., 2016; Jin et al., 2016; Hu et al., 2018; Jiao et al., 2019; Shi, 2021; Xiao, 2021; Gou, 2023), such as the random number table method. Three studies described methods for allocating concealment (Xiao, 2011; Sun et al., 2014; Jin et al., 2016), such as the sealed envelope method. None of the studies described blinding for participants or researchers. Only one study explicitly reported that its statisticians were not blinded (Jin et al., 2016), the remaining studies did not address this. All studies exhibited low loss-to-follow-up rates, with no evidence of attrition bias. No studies demonstrated selective reporting. No other biases were identified across all studies, as shown in Figures 2, 3.

### 3.4 Meta-analysis results

#### 3.4.1 Primary outcome: depression scale scores

A total of 11 RCTs were included (Xiao, 2011; Sun et al., 2014; Sun et al., 2016; Yan et al., 2015; Zhang et al., 2015; Jin et al., 2016; Hu et al., 2018; Jiao et al., 2019; Shi 2021; Xiao, 2021; Gou, 2023), involving 748 participants: 374 in the treatment group and 374 in the control group. Before intervention, the difference in depression scale scores between the treatment and control groups was not statistically significant (*p* > 0.05), demonstrating comparability between the two groups, as shown in Supplementary Figure S1.

**FIGURE 4:**
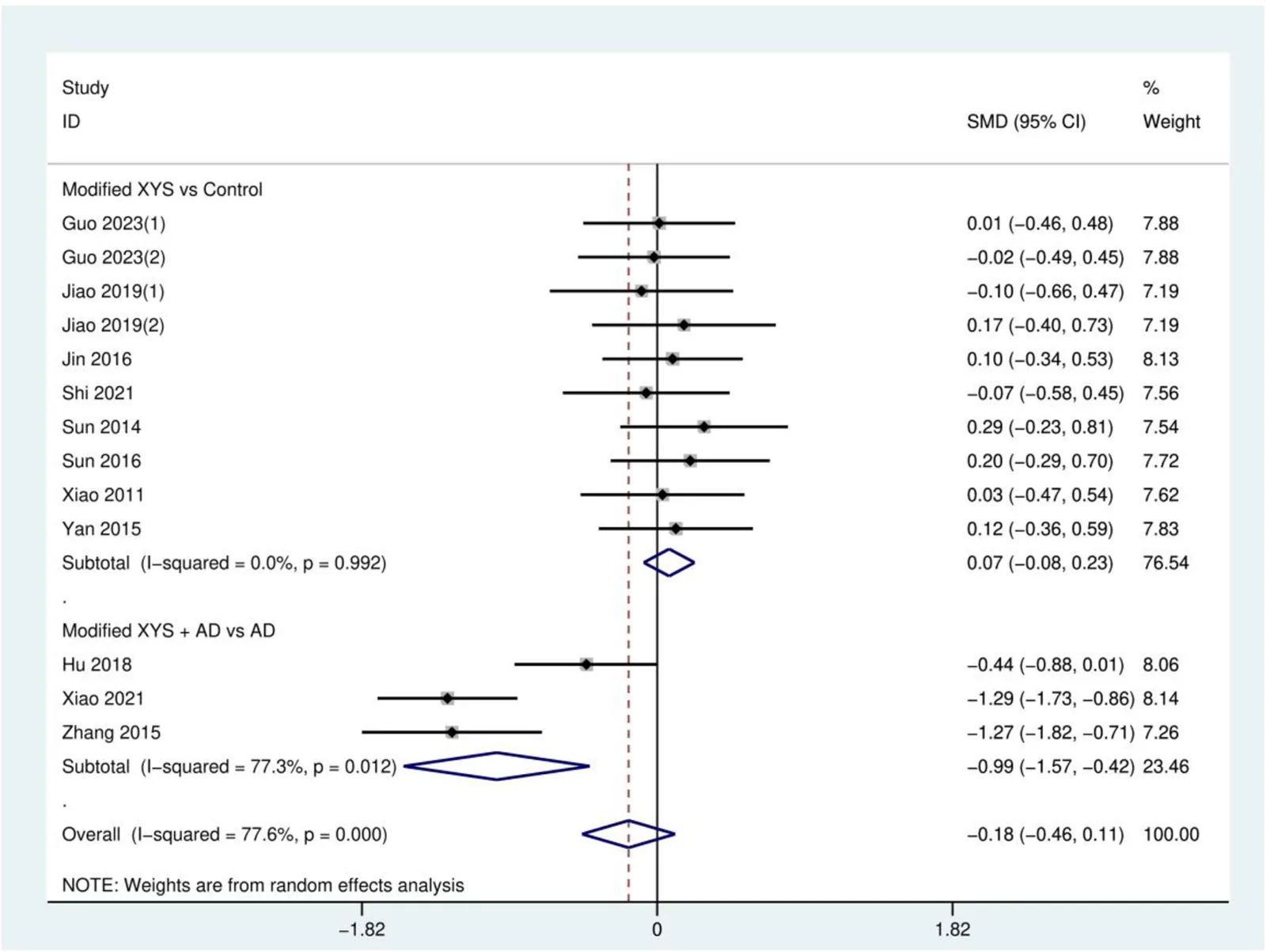
Forest plot of the effect of modified XYS on depression scale scores among postoperative depression of breast cancer patients.

In the subgroup analysis, modified XYS alone did not significantly improve depressive symptoms compared with the control group [SMD = 0.07, 95% CI (−0.08, 0.23), *p* = 0.358], and no significant heterogeneity was observed (*I* ^2^ = 0.0%, *p* = 0.992). In contrast, modified XYS combined with antidepressants (AD) significantly improved depressive symptoms compared with AD alone [SMD = −0.99, 95% CI (−1.57, −0.42), *p* = 0.001], and the heterogeneity was high (*I* ^2^ = 77.3%, *p* = 0.012). As shown in Figure 4.

To explore whether chemotherapy status contributed to clinical heterogeneity, subgroup analysis was performed according to chemotherapy status. In the chemotherapy subgroup, 5 effect estimates from RCTs were included. Modified Xiaoyao-san did not significantly improve depressive symptoms compared with control interventions [SMD = 0.05, 95% CI (−0.17, 0.28), *p* = 0.649]. No heterogeneity was observed among these studies (*I* ^2^ = 0.0%, *p* = 0.924). In the non-chemotherapy subgroup, 8 effect estimates from RCTs were included. Modified Xiaoyao-san showed a trend toward improving depressive symptoms, although the difference was not statistically significant [SMD = −0.32, 95% CI (−0.75, 0.12), *p* = 0.154]. Significant heterogeneity was observed among these studies (*I* ^2^ = 84.7%, *p* < 0.001). Overall, the pooled effect from all included RCTs showed no statistically significant difference between modified Xiaoyao-san and control interventions [SMD = −0.18, 95% CI (−0.46, 0.11), *p* = 0.229], with substantial heterogeneity (*I* ^2^ = 77.6%, *p* < 0.001). As shown in Supplementary Figure S2.

#### 3.4.2 Secondary outcome: neurotransmitters

##### 3.4.2.1 5-HT

A total of 4 RCTs were included (Sun et al., 2016; Hu et al., 2018; Xiao, 2021; Gou, 2023), involving 314 participants: 157 in the treatment group and 157 in the control group. Before intervention, the difference in 5-HT between the treatment and control groups was not statistically significant (*p* > 0.05), demonstrating comparability between the two groups, as shown in Supplementary Figure S3.

**FIGURE 5:**
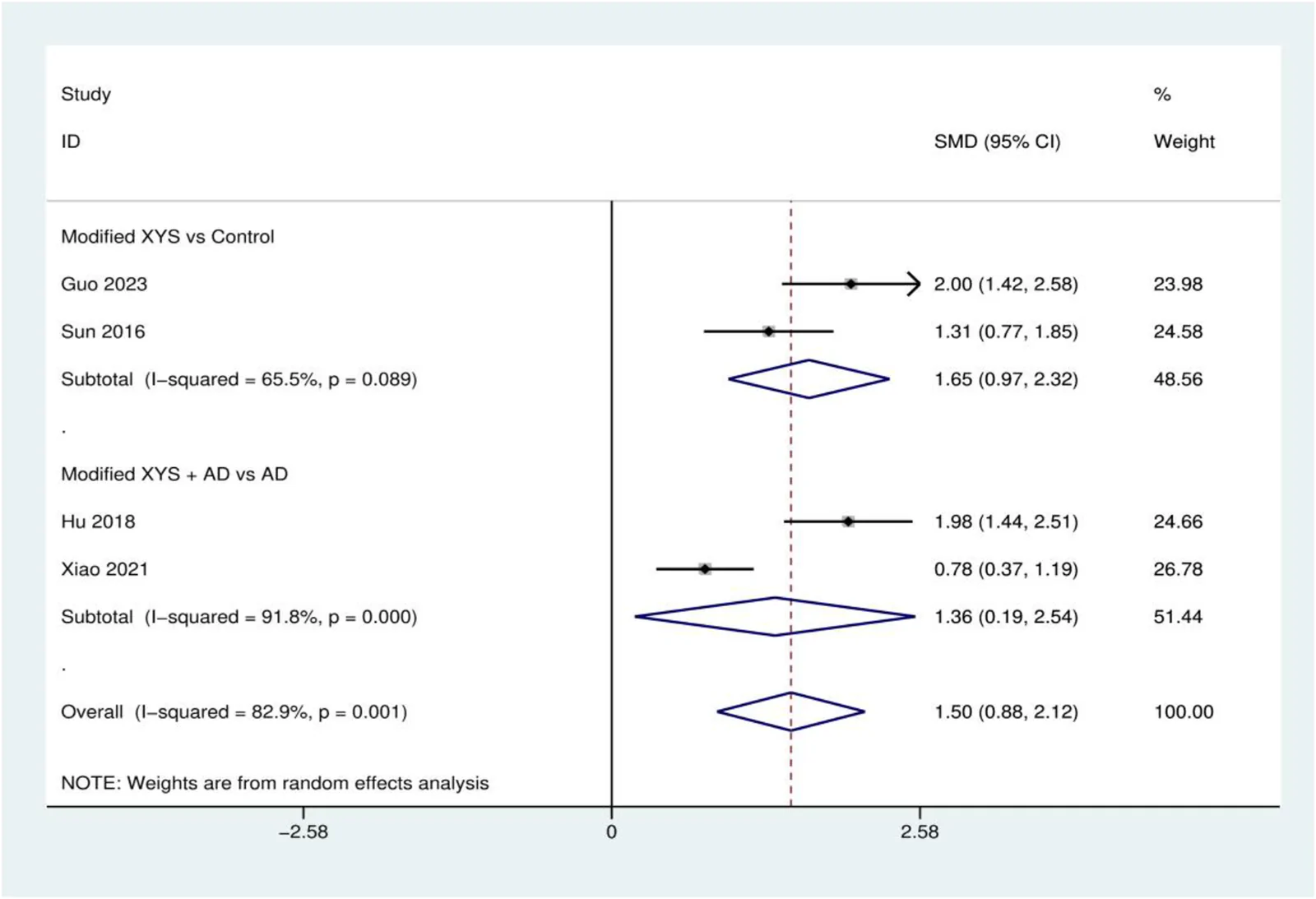
Forest plot of the effect of modified XYS on 5-HT among postoperative depression of breast cancer patients.

In the subgroup analysis, modified XYS significantly increased 5-HT levels compared with the control group [SMD = 1.65, 95% CI (0.97, 2.32), *p* < 0.001], and substantial heterogeneity was observed (*I* ^2^ = 65.5%, *p* = 0.089). In addition, modified XYS plus AD significantly increased 5-HT levels compared with AD alone [SMD = 1.36, 95% CI (0.19, 2.54), *p* = 0.023], and high heterogeneity was observed (*I* ^2^ = 91.8%, *p* < 0.001). As shown in Figure 5.

##### 3.4.2.2 DA

A total of 3 RCTs were included (Sun et al., 2016; Hu et al., 2018; Xiao, 2021), involving 244 participants: 122 in the treatment group and 122 in the control group. Before intervention, the difference in DA between the treatment and control groups was not statistically significant (*p* > 0.05), demonstrating comparability between the two groups, as shown in Supplementary Figure S4.

**FIGURE 6:**
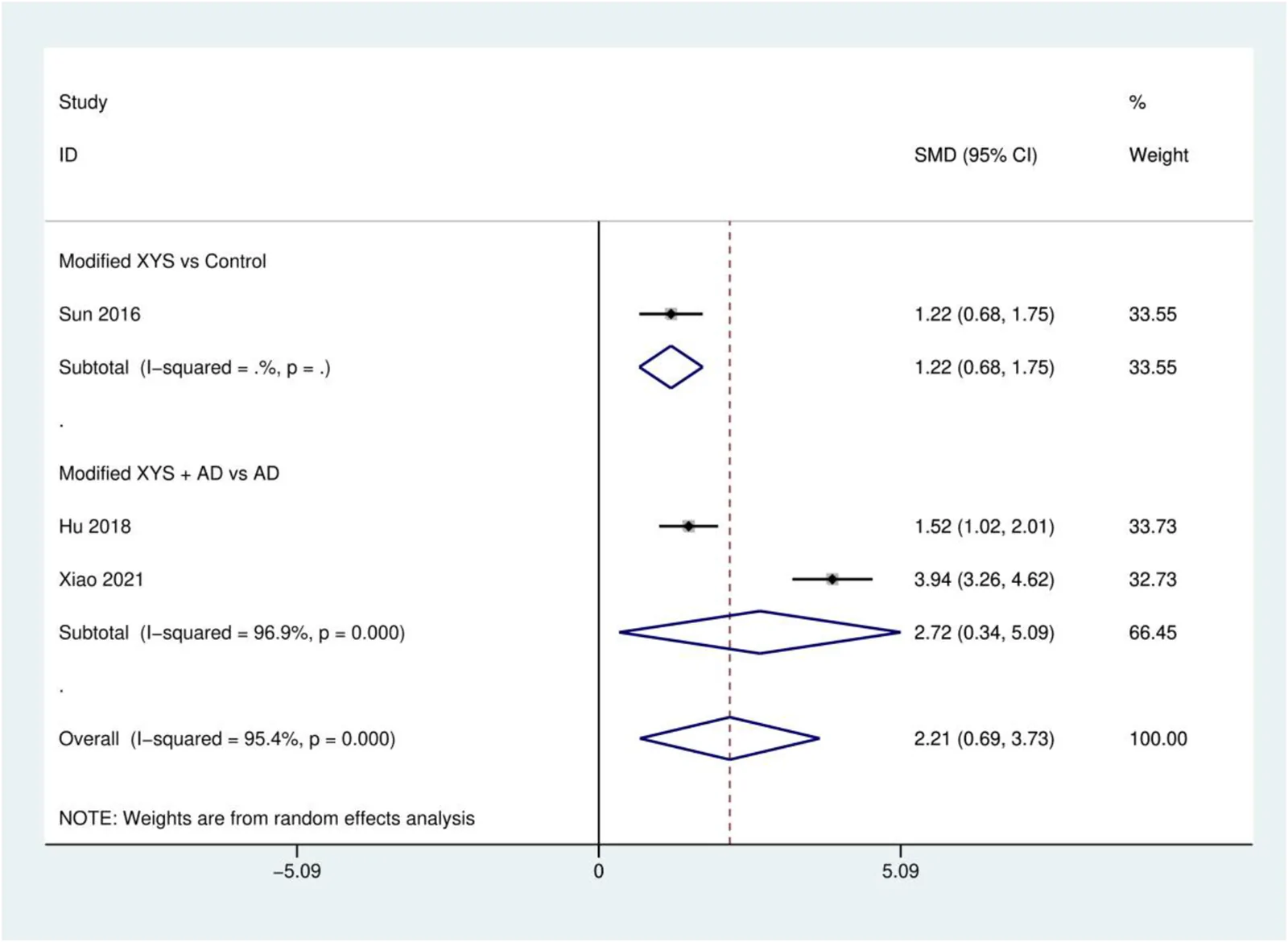
Forest plot of the effect of modified XYS on DA among postoperative depression of breast cancer patients.

In the subgroup analysis, modified XYS significantly increased DA levels compared with the control group [SMD = 1.22, 95% CI (0.68, 1.75), *p* < 0.001], and heterogeneity was not applicable because only one study was included. In addition, modified XYS plus AD significantly increased DA levels compared with AD alone [SMD = 2.72, 95% CI (0.34, 5.09), *p* = 0.025], and high heterogeneity was observed (*I* ^2^ = 96.9%, *p* < 0.001). As shown in Figure 6.

##### 3.4.2.3 NE

A total of 4 RCTs were included (Sun et al., 2016; Hu et al., 2018; Xiao, 2021; Gou, 2023), involving 314 participants: 157 in the treatment group and 157 in the control group. Before intervention, the difference in NE between the treatment and control groups was not statistically significant (*p* > 0.05), demonstrating comparability between the two groups, as shown in Supplementary Figure S5.

**FIGURE 7:**
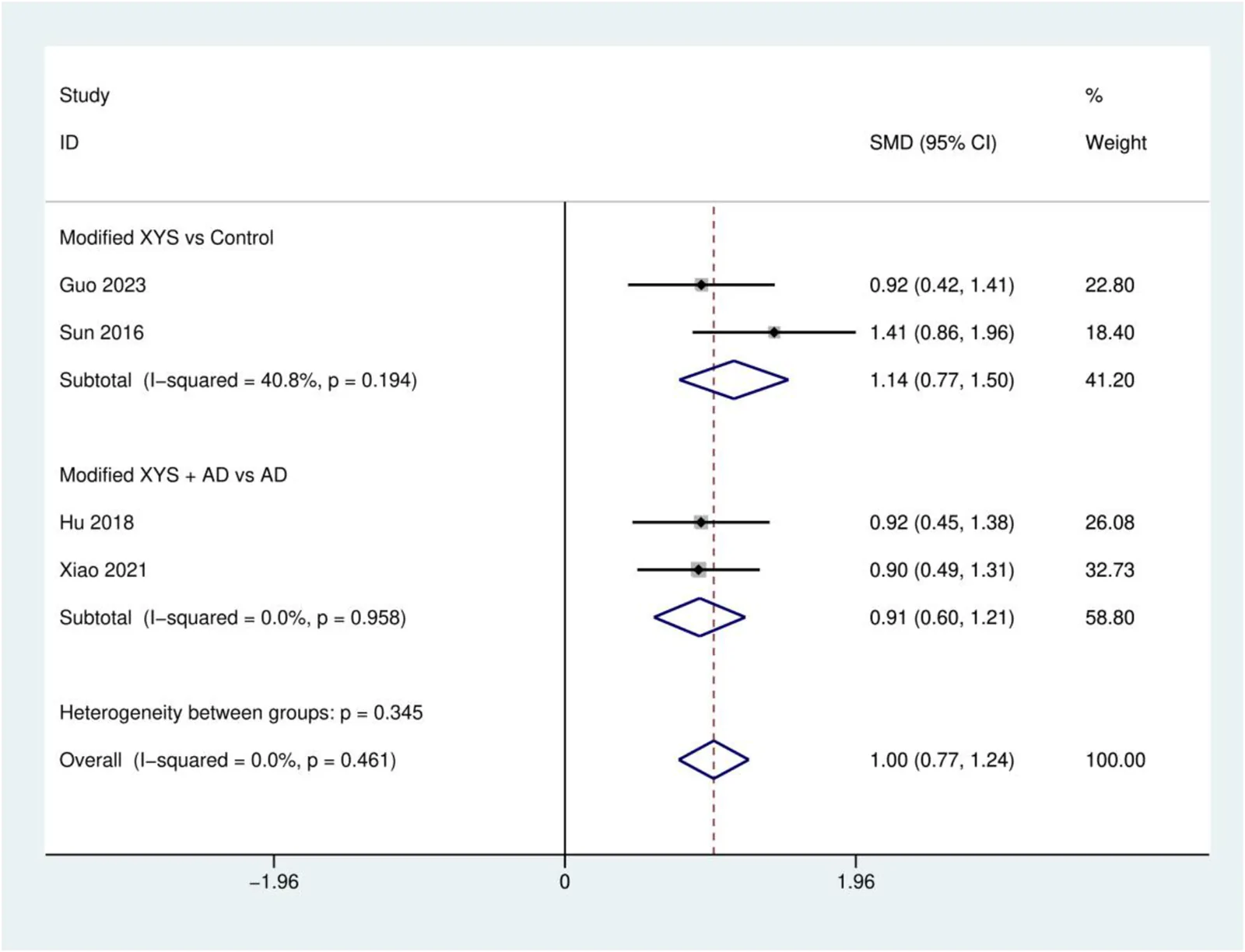
Forest plot of the effect of modified XYS on NE among postoperative depression of breast cancer patients.

In the subgroup analysis, modified XYS significantly increased NE levels compared with the control group [SMD = 1.14, 95% CI (0.77, 1.50), *p* < 0.001], and slight heterogeneity was observed (*I* ^2^ = 40.8%, *p* = 0.194). In addition, modified XYS plus AD significantly increased NE levels compared with AD alone [SMD = 0.91, 95% CI (0.60, 1.21), *p* < 0.001], and no significant heterogeneity was observed (*I* ^2^ = 0.0%, *p* = 0.958). As shown in Figure 7.

### 3.5 Sensitivity analysis

**FIGURE 8:**
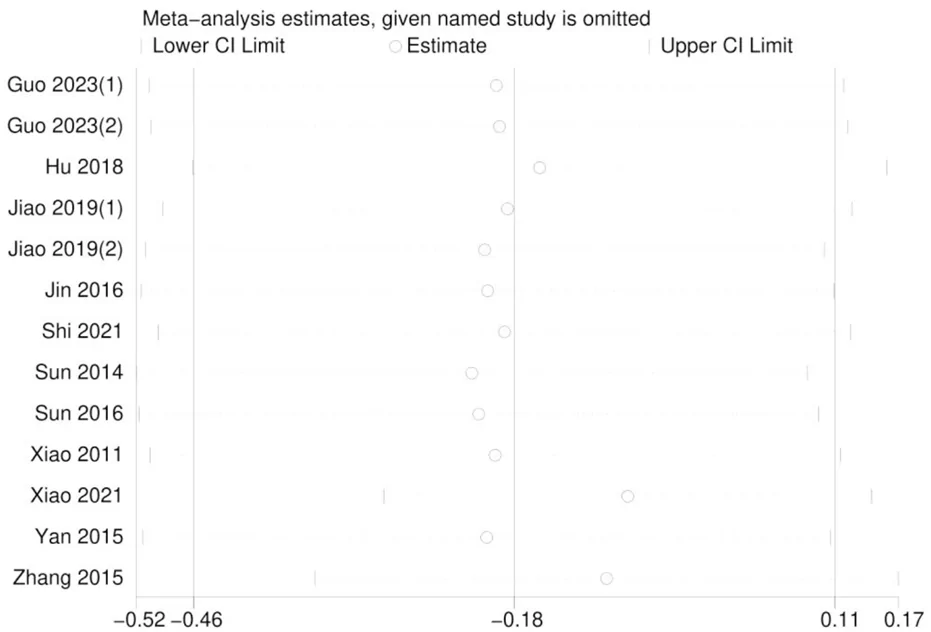
Sensitivity analysis of the primary outcome.

Sensitivity analysis was conducted on the primary outcome, as shown in Figure 8. Results indicate that the pooled effect size remained within the 95% CI even after excluding each individual study. This demonstrates that the findings of this study are reliable and robust.

### 3.6 Publication bias analysis

**FIGURE 9:**
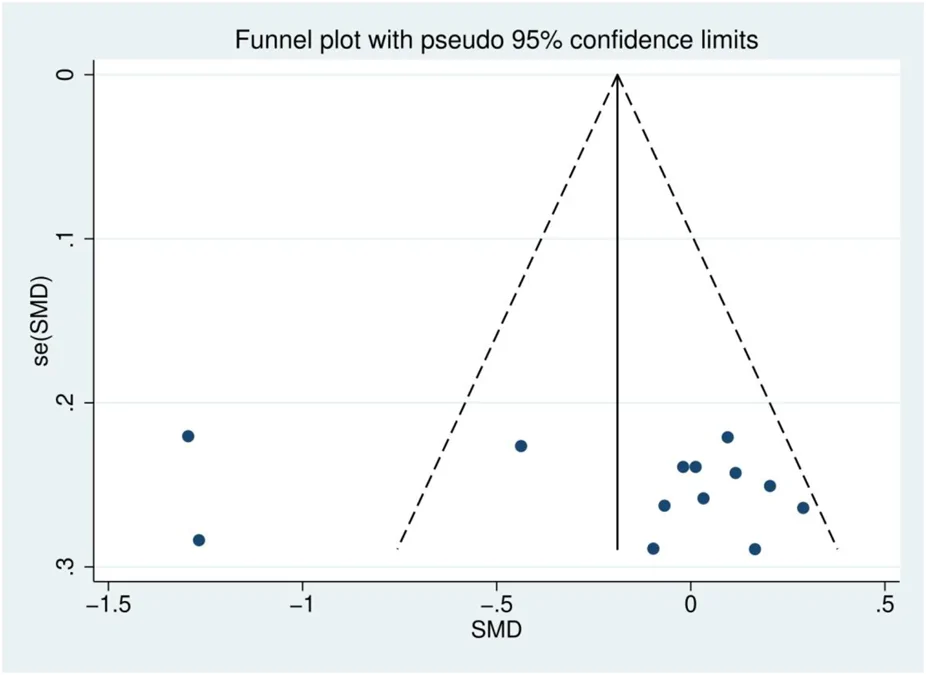
Funnel plot of primary outcome.

**FIGURE 10:**
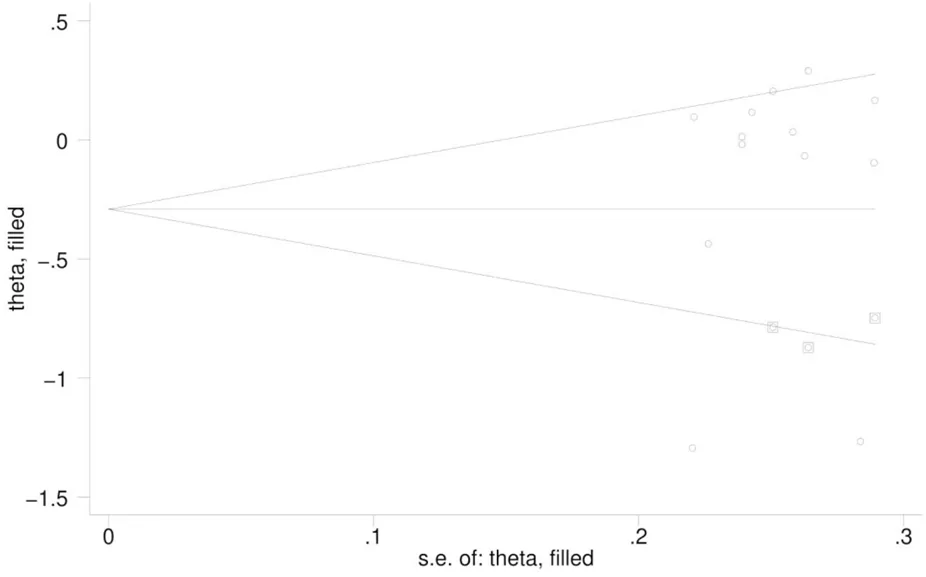
Trim and fill adjustment of the primary outcome.

Funnel plots, Egger’s test, and Begg’s test were used to assess publication bias. The funnel plot revealed slight asymmetry, as shown in Figure 9. Additionally, Egger’s test yielded a *p*-value less than 0.05 (*p* = 0.567), while Begg’s test produced a *p*-value greater than 0.05 (*p* = 0.669), indicating the presence of publication bias, as depicted in Supplementary Figure S6. To assess the impact of publication bias on this study, we employed trim-and-fill adjustment, as shown in Figure 10. The unadjusted depression scale scores were [SMD = −0.18, 95% CI (−0.46, 0.11), *p* < 0.001]. The adjusted depression scale scores after trim-and-fill adjustment were [SMD = −0.29, 95% CI (−0.55, −0.03), *p* < 0.001], indicating that despite publication bias, the adjusted results supported the initial conclusion, as shown in Supplementary Figure S7.

## 4 Discussion

### 4.1 Mechanism of action of modified XYS on postoperative depression of breast cancer

Breast cancer surgery severely consumes qi and blood and damages visceral function. Physical trauma together with postoperative anxiety leads to liver qi stagnation. Stagnated liver qi overrestrains the spleen, resulting in dysfunction of transportation and transformation. This progressive pathological evolution constitutes the core syndrome of liver stagnation, blood deficiency and spleen deficiency in patients with postoperative depression. Under the guidance of treatment based on syndrome differentiation, XYS can soothe the liver, invigorate the spleen and nourish blood simultaneously, which precisely addresses both liver-spleen disharmony and qi-blood deficiency caused by surgical trauma. Clinically, surgical damage often produces various accompanying syndromes. Long-term qi stagnation will develop into blood stasis, so blood-activating and stasis-resolving botanical drugs are supplemented. Excessive loss of body fluid during the operation easily causes yin deficiency, so yin-nourishing medicines are added to replenish consumed yin fluid. Severe emotional fluctuation disturbs the heart spirit, hence tranquilizing botanical drugs are used to stabilize the mind. All modified prescriptions retain the core botanical drug composition of XYS and adjust auxiliary medicines according to the secondary syndromes caused by surgery. This flexible compatibility fully reflects the holistic treatment and syndrome modification principles of traditional Chinese medicine.

Research has demonstrated that modified XYS exerts its antidepressant effects through multi-target, multi-pathway systemic regulation. It intervenes in core processes such as neuroinflammation, apoptosis, and neuroplasticity (Jiao et al., 2024; Zhang W. et al., 2024), thereby achieving holistic balance of emotional and neural functions at the central nervous system level. Mechanistically, modified XYS effectively suppresses neuroinflammatory responses and abnormal cytokine release by modulating the TRIM31/NLRP3 inflammasome pathway (Wang et al., 2024). Additionally, modified XYS modulates the hippocampal PI3K/Akt signaling pathway to mitigate glutamate excitotoxicity and promote neuronal repair (Zhou et al., 2021). The synergistic interaction between these two mechanisms collectively maintains neural network homeostasis, promotes neuroplasticity reconstruction, and mitigates stress-induced damage, thereby achieving a holistic antidepressant effect. Additionally, modified XYS can remodel the gut microbiota to reduce lipopolysaccharide (LPS) production and systemic circulation, thereby suppressing LPS/TLR4-mediated neuroinflammation in the brain and ultimately exerting antidepressant effects (Zhu et al., 2019; Liu et al., 2024).

Notably, existing research suggests that modified XYS may also exert antitumor proliferation and metastasis effects by downregulating the expression of Bcl-2 protein in breast cancer and upregulating molecules such as Bax and p53 (Chen et al., 2012). Although the primary objective of this study was to investigate the antidepressant efficacy of modified XYS among postoperative depression of breast cancer patients, these findings suggest that its application in breast cancer patients may offer potential oncological benefits and demonstrate safety, thereby providing a solid theoretical foundation for its clinical implementation.

### 4.2 Comparison with previous studies

Compared with previous studies, this research highlights its unique value in terms of disease types and intervention measures. 1) In terms of disease type, this study focuses on postoperative depression of breast cancer, whose etiology is closely linked to cancer diagnosis, surgical trauma, and subsequent treatment. This differs from Liu et al.‘s research on postpartum depression associated with perinatal hormonal fluctuations (Liu et al., 2025), as well as from Wang et al.‘s study on post-stroke depression directly caused by cerebrovascular events (Wang X.-L. et al., 2022). This fundamental difference in pathogenesis may lead to distinct therapeutic emphases for modified XYS across different diseases. 2) Regarding the intervention, unlike the evaluation of the efficacy of all traditional Chinese medicine therapies for postoperative depression of breast cancer patients (Wang et al., 2023), this study specifically limited the intervention to the classical formula modified XYS. By focusing on a single, well-defined intervention within the same disease context, the study yielded more targeted and clinically relevant conclusions, thereby deepening our understanding of modified XYS’s therapeutic efficacy. In summary, this study emphasizes that modified XYS is an effective treatment for postoperative depression of breast cancer. This helps to expand the alternative treatment of cancer-related depression, and provides a reference for future evidence-based research in this field.

### 4.3 Safety profile and potential interactions of modified XYS

The safety profile of modified XYS is an important consideration for its clinical application. Since modified XYS is derived from XYS, previous evidence regarding XYS provides preliminary information regarding the safety profile of modified XYS. Previous systematic reviews and meta-analyses of XYS reported that adverse events were generally mild, including nausea, dry mouth, headache, dizziness, and somnolence (Wang et al., 2025). Furthermore, these systematic reviews and meta-analyses suggested that XYS was associated with a lower incidence of adverse events compared with conventional treatments, indicating acceptable tolerability (Xiong et al., 2019). However, evidence regarding the interactions of modified XYS remains limited. Future clinical studies should incorporate standardized adverse event monitoring, longer follow-up periods, and systematic evaluation of potential interactions with concomitant medications to further establish the safety profile of modified XYS.

### 4.4 Limitations

This study has several limitations that require improvement in future research. First, all included studies originated from China, lacking multinational and multiethnic data support. Therefore, international multicenter studies should be conducted to validate its universality across different cultural and genetic backgrounds. Second, the modified XYS formulae used across studies varied in dosage, lacking a standardized intervention protocol. Future efforts should establish a standardized formulation based on evidence-based medicine to enhance research consistency. Third, some included trials lacked clear descriptions of allocation concealment and blinding procedures, which may introduce selection and performance bias and potentially overestimate therapeutic effects. Higher-quality clinical trials are urgently needed to explore its long-term efficacy. Fourth, the original trials did not track adverse events systematically, so we cannot evaluate the long-term safety of medication. Higher-quality clinical trials are still needed in the future to clarify its medication risks.

## 5 Conclusion

Available evidence suggests that modified XYS exerts positive effects on depression scale scores, neurotransmitters among postoperative depression of breast cancer patients. However, due to the limited number of included studies, further validation requires additional high-quality RCTs.

## Data Availability

The original contributions presented in the study are included in the article/Supplementary Material, further inquiries can be directed to the corresponding author.

## References

[B1] Aapro M. Cull A. (1999). Depression in breast cancer patients: the need for treatment. Ann. Oncol. 10, 627–636. 10.1023/a:1008328005050 10442183

[B2] Ahn J. Suh E. E. (2023). Body image alteration in women with breast cancer: a concept analysis using an evolutionary method. Asia Pac J. Oncol. Nurs. 10, 100214. 10.1016/j.apjon.2023.100214 37213808 PMC10199402

[B3] Begg C. B. Mazumdar M. (1994). Operating characteristics of a rank correlation test for publication bias. Biometrics 50, 1088–1101. 7786990

[B4] Bray F. Laversanne M. Sung H. Ferlay J. Siegel R. L. Soerjomataram I. (2024). Global cancer statistics 2022: GLOBOCAN estimates of incidence and mortality worldwide for 36 cancers in 185 countries. CA Cancer J. Clin. 74, 229–263. 10.3322/caac.21834 38572751

[B5] Chen W.-F. Xu L. Yu C.-H. Ho C.-K. Wu K. Leung G. C. (2012). The *in vivo* therapeutic effect of free wanderer powder (Xiāo Yáo Sǎn, Xiaoyaosan) on mice with 4T1 cell induced breast cancer model. J. Tradit. Complement. Med. 2, 67–75. 10.1016/s2225-4110(16)30073-6 24716117 PMC3943014

[B6] Czajka M. L. Pfeifer C. (2025). Breast cancer surgery,” in Statpearls. Treasure Island (FL): StatPearls Publishing. Available online at: http://www.ncbi.nlm.nih.gov/books/NBK553076/ (Accessed September 02, 2025). 31971717

[B7] Duval S. Tweedie R. (2000). Trim and fill: a simple funnel-plot-based method of testing and adjusting for publication bias in meta-analysis. Biometrics 56, 455–463. 10.1111/j.0006-341x.2000.00455.x 10877304

[B8] Egger M. Davey Smith G. Schneider M. Minder C. (1997). Bias in meta-analysis detected by a simple, graphical test. BMJ 315, 629–634. 10.1136/bmj.315.7109.629 9310563 PMC2127453

[B9] Golden-Kreutz D. M. Andersen B. L. (2004). Depressive symptoms after breast cancer surgery: relationships with global, cancer-related, and life event stress. Psychooncology 13, 211–220. 10.1002/pon.736 15022156 PMC2150738

[B10] Gou S. Q. (2023). Clinical study on the effect of modified Xiaoyao-san in treating depression syndrome in postoperative breast cancer patients with chemotherapy-induced liver depression and spleen deficiency. Mod. Med. Health Res. Electron. J. 7, 79–81.

[B11] Hu X. Li F. Z. Shang Y. P. Wang N. Han T. Li J. (2018). Effect of jiawei xiaoyao pill on plasma 5-HT, DA and NE in depression after breast cancer surgery. J. Changchun Univ. Chin. Med. 34, 725–727. 10.13463/j.cnki.cczyy.2018.04.035

[B12] Javan Biparva A. Raoofi S. Rafiei S. Masoumi M. Doustmehraban M. Bagheribayati F. (2023). Global depression in breast cancer patients: systematic review and meta-analysis. PLoS One 18, e0287372. 10.1371/journal.pone.0287372 37494393 PMC10370744

[B13] Jiao J. Luo K. H. Li Y. H. Li D. F. Tang J. Y. (2019). Clinical observation on modified xiaoyao powder treatment for patients with depression syndrome of liver depression and spleen deficiency during adjuvant chemotherapy after breast cancer operation. Anti-tumor Pharm. 9, 107–111. 10.3969/j.issn.2095-1264.2019.01.23

[B14] Jiao H. Fan Y. Gong A. Li T. Fu X. Yan Z. (2024). Xiaoyaosan ameliorates CUMS-Induced depressive-like and anorexia behaviors in mice *via* necroptosis related cellular senescence in hypothalamus. J. Ethnopharmacol. 318, 116938. 10.1016/j.jep.2023.116938 37495029

[B15] Jin J. J. Yu Z. Y. Xu B. (2016). Clinical observation on fuzheng jieyu decoction in treatment of hepatic stagnation and spleen deficiency type depression after breast cancer operation. China Mod. Dr. 54, 123–126.

[B16] Liu X. Liu H. Wu X. Zhao Z. Wang S. Wang H. (2024). Xiaoyaosan against depression through suppressing LPS mediated TLR4/NLRP3 signaling pathway in “microbiota-gut-brain” axis. J. Ethnopharmacol. 335, 118683. 10.1016/j.jep.2024.118683 39121928

[B17] Liu J. Rong A. Wang F. (2025). Meta-analysis of xiaoyao formula as an adjuvant therapy for treating postpartum depression. Front. Psychiatry 16, 1558505. 10.3389/fpsyt.2025.1558505 40195969 PMC11973275

[B18] Navari R. M. Brenner M. C. Wilson M. N. (2008). Treatment of depressive symptoms in patients with early stage breast cancer undergoing adjuvant therapy. Breast Cancer Res. Treat. 112, 197–201. 10.1007/s10549-007-9841-z 18064563

[B19] Page M. J. McKenzie J. E. Bossuyt P. M. Boutron I. Hoffmann T. C. Mulrow C. D. (2021). The PRISMA 2020 statement: an updated guideline for reporting systematic reviews. BMJ 372, n71. 10.1136/bmj.n71 33782057 PMC8005924

[B20] Pan J. Fu S. Zhou Q. Lin D. Chen Q. (2023). Modified xiaoyao san combined with chemotherapy for breast cancer: a systematic review and meta-analysis of randomized controlled trials. Front. Oncol. 13, 1050337. 10.3389/fonc.2023.1050337 37035186 PMC10073574

[B21] Phoosuwan N. Lundberg P. C. (2023). Life satisfaction, body image and associated factors among women with breast cancer after mastectomy. Psychooncology 32, 610–618. 10.1002/pon.6106 36670514

[B22] Pilevarzadeh M. Amirshahi M. Afsargharehbagh R. Rafiemanesh H. Hashemi S.-M. Balouchi A. (2019). Global prevalence of depression among breast cancer patients: a systematic review and meta-analysis. Breast Cancer Res. Treat. 176, 519–533. 10.1007/s10549-019-05271-3 31087199

[B23] Setyowibowo H. Yudiana W. Hunfeld J. A. M. Iskandarsyah A. Passchier J. Arzomand H. (2022). Psychoeducation for breast cancer: a systematic review and meta-analysis. Breast 62, 36–51. 10.1016/j.breast.2022.01.005 35121502 PMC8819101

[B24] Shi W. X. (2021). Observation on the Effect of Xiaoyao Powder and Kaixin Powder in the Prevention and Treatment of tumor-related Depression of HER-2 Positive Breast Cancer. Jinan, Shandong Province: Shandong University of Traditional Chinese Medicine. 10.27282/d.cnki.gsdzu.2021.000484

[B25] Sterne J. A. C. Savović J. Page M. J. Elbers R. G. Blencowe N. S. Boutron I. (2019). RoB 2: a revised tool for assessing risk of bias in randomised trials. BMJ 366, l4898. 10.1136/bmj.l4898 31462531

[B26] Sun S. L. Zhang H. R. Yang L. P. Zhang Y. H. (2014). Clinical research on modified xiaoyaosan in treating breast cancer postoperative depression. China J. Chin. Med. 29, 1708–1709. 10.16368/j.issn.1674-8999.2014.12.063

[B27] Sun S. L. Yang L. P. Zhang H. R. Wu Y. S. (2016). Effects of modified xiaoyao powder on breast cancer patients with depressive disorder treated with chemotherapy after operation. China J. Traditional Chin. Med. Pharm. 31, 1499–1502.

[B28] Wang D. Li T. Ron J. Mao M. Yang Y. Feng Y. (2022). Efficacy and safety of xiaoyao recipe in the treatment of poststroke depression: a systematic review and meta-analysis. Evidence-Based Complement. Altern. Med. 2022, 1–10. 10.1155/2022/4385783 35463080 PMC9020944

[B29] Wang Y. Liu S. Zhang Y. Zhu G. Wang H. Xu B. (2023). Effect of traditional Chinese medicine on postoperative depression of breast cancer: a systematic review and meta-analysis. Front. Pharmacol. 14, 1019049. 10.3389/fphar.2023.1019049 37426820 PMC10327430

[B30] Wang B. Tian L. Wu M. Zhang D. Yan X. Bai M. (2024). Modified danzhi XiaoyaoSan inhibits neuroinflammation via regulating TRIM31/NLRP3 inflammasome in the treatment of CUMS depression. Exp. Gerontol. 192, 112451. 10.1016/j.exger.2024.112451 38729250

[B31] Wang Q. Zhou J. Gong G. (2025). Efficacy and safety of xiaoyao san in the treatment of chronic fatigue syndrome: a systematic review and meta-analysis. Front. Pharmacol. 16, 1496774. 10.3389/fphar.2025.1496774 39981187 PMC11839657

[B32] Wang X.-L. Feng S.-T. Wang Y.-T. Zhang N.-N. Wang Z.-Z. Zhang Y. (2022). Canonical Chinese medicine formula Danzhi-Xiaoyao-San for treating depression: a systematic review and meta-analysis. J. Ethnopharmacol. 287, 114960. 10.1016/j.jep.2021.114960 34968660

[B33] Xiao B. (2011). The Depression of Breast Cancer: Analysis of Etiology, Pathogenesis and Treatments Based on Syndrome Differentiation in Traditional Chinese Medicine. Guangzhou, Guangdong: Guangzhou University of Chinese Medicine. Available online at: https://kns.cnki.net/KCMS/detail/detail.aspx?dbcode=CMFD&dbname=CMFD2011&filename=1011135427.nh (Accessed October 25, 2025).

[B34] Xiao F. (2021). Clinical efficacy of modified xiaoyao powder combined with paroxetine in the adjuvant treatment of postoperative depression in breast cancer patients. Chin. J. Clin. Ration. Drug Use 14, 78–80. 10.15887/j.cnki.13-1389/r.2021.13.031

[B35] Xiong X. J. Wang P. Q. Duan L. Liu W. Chu F. Y. Li S. J. (2019). Efficacy and safety of Chinese herbal medicine xiao Yao san in hypertension: a systematic review and meta-analysis. Phytomedicine 61, 152849. 10.1016/j.phymed.2019.152849 31035044

[B36] Yan Y. J. Fu W. Zhou J. C. Hu L. T. Fan W. T. (2015). Modified xiaoyao powder in treatment of postoperative breast cancer accompanied with depression. J. Changchun Univ. Chin. Med. 31, 781–782. 10.13463/j.cnki.cczyy.2015.04.045

[B37] Zhang L. H. Zhuang Z. J. Wang J. C. (2015). Clinical research on the treatment of postoperative breast cancer with depression by modified xiaoyao powder combined with fluoxetine hydrochloride capsules. China J. Chin. Med. 30, 785–787. 10.16368/j.issn.1674-8999.2015.06.271

[B38] Zhang Q. Wu G. Chen J. Fang K. Liu Q. Zhang P. (2024). Factors influencing depressive symptoms in Chinese female breast cancer patients: a meta-analysis. Front. Psychol. 15, 1332523. 10.3389/fpsyg.2024.1332523 38659682 PMC11039958

[B39] Zhang W. Guo Z. Wang Y. Fang S. Wan C. Yu X. (2024). From perspective of hippocampal plasticity: function of antidepressant Chinese medicine xiaoyaosan. Chin. J. Integr. Med. 30, 747–758. 10.1007/s11655-024-3908-0 38900227

[B40] Zhou X.-M. Liu C.-Y. Liu Y.-Y. Ma Q.-Y. Zhao X. Jiang Y.-M. (2021). Xiaoyaosan alleviates hippocampal glutamate-induced toxicity in the CUMS rats via NR2B and PI3K/Akt signaling pathway. Front. Pharmacol. 12, 586788. 10.3389/fphar.2021.586788 33912031 PMC8075411

[B41] Zhu H.-Z. Liang Y.-D. Ma Q.-Y. Hao W.-Z. Li X.-J. Wu M.-S. (2019). Xiaoyaosan improves depressive-like behavior in rats with chronic immobilization stress through modulation of the gut microbiota. Biomed. and Pharmacother. 112, 108621. 10.1016/j.biopha.2019.108621 30798141

